# Self-Medication Practices among Adult Population in Bangladesh: A Cross-Sectional Study

**DOI:** 10.3390/epidemiologia5020010

**Published:** 2024-04-01

**Authors:** Nitai Roy, Md. Nazrul Islam, Md. Shahjalal, Aysha Siddiky, Sultan Mahmud Imran, Md. Aktarujjaman, Md. Mobarak Hossain, Bryan T. Rogers, Kamal Krishna Biswas, Ekhtear Hossain

**Affiliations:** 1 Department of Biochemistry and Food Analysis, Patuakhali Science and Technology University, Patuakhali 8602, Bangladesh; 2 Department of Post-Harvest Technology and Marketing, Patuakhali Science and Technology University, Patuakhali 8602, Bangladesh; 3 Department of Public Health, North South University, Dhaka 1229, Bangladesh; 4 Research Rats, Dhaka 1229, Bangladesh; 5 Faculty of Nutrition and Food Science, Patuakhali Science and Technology University, Patuakhali 8602, Bangladesh; 6 Department of Biological Sciences and Chemistry, Southern University and A&M College, Baton Rouge, LA 70813, USA; bryan_rogers@subr.edu; 7 Department of Biochemistry and Molecular Biology, University of Rajshahi, Rajshahi 6205, Bangladesh

**Keywords:** self-medication, prevalence, risk factors, Bangladesh

## Abstract

Background: The practice of self-medication (SM) is common worldwide and is an important component of medical self-care. However, improper practice can be dangerous. This study aimed to estimate the prevalence of SM and the factors associated with it among Bangladeshi adults. Methods: A cross-sectional survey was conducted between April and June 2021 among Bangladeshi adults (aged > 19 years) using convenient sampling. A total of 1320 subjects were collected through face-to-face interviews using a standardized questionnaire. Multivariable logistic regression analysis was used to identify factors associated with the practice of SM. Results: Overall, 41% of adults in our survey reported SMP. The most common illnesses that prompted SM were common cold/flu (66.4%), gastric problems (65%), and headache (64.4%). The most frequent reasons for SM were to get better-perceived quality of care (30.6%), perceiving SM without side effects (23.3%), and saving time with effectiveness (14.56%). Potential risk factors included 10 years (AOR = 1.91; 95% CI: 1.04–3.50) and >12 years of schooling (AOR = 5.03; 95% CI: 2.27–11.15), being a businessman (AOR = 4.64; 95% CI: 1.74–12.37), having ≤6 family members (AOR = 2.13; 95% CI: 1.40–3.24), being a member of a social group (AOR = 1.53; 95% CI: 1.10–2.12), a health status check after every six months (AOR = 1.52; 95% CI: 1.08–2.13), and current ill-health (AOR = 1.41; 95% CI: 1.06–1.87). Protective factors identified included ≤30 years of age (AOR = 0.40; 95% CI: 0.17–0.93), and practice of modern (AOR = 0.39; 95% CI: 0.22–0.69) and herbal (AOR = 0.45; 95% CI: 0.21–0.97) treatment modality. Conclusion: More than one-third of the study participants reported practicing SM. Increasing the community’s awareness of the adverse outcomes of SM and not just the average experience might sway individuals away from SM, and implementing strict jurisdiction could be a way to minimize inappropriate SM.

## 1. Introduction

The World Health Organization (WHO) defines self-medication (SM) as when a person takes medications based on their self-diagnosis of an illness without going to a doctor or doing any clinical tests to support their beliefs [1]. SM encompasses not only taking medications for acute symptoms but also repeatedly administering medications for chronic illnesses [2]. The practice of SM, selecting and ingesting medications without consulting a physician for disease prevention, diagnosis, or treatment, is a worldwide phenomenon [3]. In 2013, it was estimated that 246 million people, or one in every 20 people aged 15–64 years worldwide, had self-medicated [4]. Insufficient drug regulation, the easy availability of over-the-counter medications, and unequal distribution of healthcare have been shown to contribute to the prevalence of this practice [5], while additional factors, such as direct marketing of drugs to the public, are also suspected.

SM is common across nations independent of developmental or economic rank. For example, in the West, 70.1% of Americans [6], 16.1% of Brazilians [7], and 69.2% of Italians [8] self-medicate. Meanwhile, in Asia, 32.5% of Indians [9], 84.8% of Pakistanis [10], and 38.2% of Nepalis [11] self-medicate. In Bangladesh, a recent study found that SM is common among the higher-educated population in metropolitan areas (16–81%) [12]. Another study carried out in Bangladesh’s Comilla area found that 73.6% of adults regularly self-medicated, and they did so specifically for mild illnesses due to their familiarity with the medications [13]. According to research among Savar residents in Bangladesh, pharmacy outlets, prior experience or prescriptions, and peer (friend or relative) consultation were the most common influences on SM, with a reported incidence of 60.2% [14]. In a cross-sectional study on Bangladeshi people’s knowledge, attitudes, and awareness of SM, 80% of respondents admitted to buying or using medications without a prescription. Socioeconomic and lifestyle factors were the leading influences on this behavior [15].

The self-reported reasons for choosing SM are varied and include insufficient time to consult with medical personnel, inability to schedule a quick appointment, long distances and travel times to reach the nearest hospitals and clinics, limited openings for immediate treatment from a hospital during busy hours, lack of healthcare services, and unaffordable consulting fees for medical doctors [16]. Studies conducted in Europe [17], South West Ethiopia [18], and Bangladesh [19] show that individuals choose SM for common ailments like fever, cold, acidity, abdominal pain, cough, headache, back pain, typhoid, typhus, cough, community-acquired pneumonia, diarrhea, amebiasis, tonsillitis, wounds, allergies, and diarrhea.

Healthcare systems have become increasingly expensive, and developing nations typically have limited healthcare facilities, making SM an obvious choice for healthcare services at that time. The individual benefits of SM are cost savings, especially considering that medical consultations will be reduced or eliminated [1], convenience, and the agency provided by participation in one’s own health care. At the community level, the benefits include avoiding the waste of scarce medical resources for minor conditions, reducing the costs of programs funded by the community for healthcare, reducing the burden on medical services in areas with inadequate healthcare personnel, and increasing the availability of affordable healthcare for rural and remote populations [1]. Thus, there are many situations where SM is a reasonably safe alternative to professionally managed care.

However, despite the evident advantages, SM can have detrimental consequences to both individual and community health [20]. SM may result in health hazards such as adverse drug reactions [20], inaccurate or delayed diagnosis, and extended suffering associated with the disease [21]. As in other countries, Bangladesh has experienced high levels of bacterial resistance to widely and overused antimicrobials resulting from SM [22]. This means that higher rates of resistant bacterial infections continue because antibiotics are not administered appropriately in the context of self-administration practices [21,23]. Antimicrobial resistance is thought to be at the heart of up to 80% of infections-related fatalities, prompting a recent petition to the Bangladesh High Court seeking a ruling barring the sale of drugs without moral justification [24].

Improving individual and community health care requires understanding the common practices, reasons for, and factors that impact SM. Adult behavior has a significant impact on household and community health choices. Understanding the reasons for SM can help shape policies that encourage safe self-care habits, leading to better public health for adults. Unfortunately, minimal information is available on the prevalence of practicing SM among adults and its associated factors in Bangladesh. Therefore, the study aimed to explore (i) the prevalence of SM among Bangladeshi adults and (ii) the associated factors leading to SM.

## 2. Methods

### 2.1. Study Design and Period

A cross-sectional study was carried out to investigate the prevalence, indications, disease conditions, presence or absence of adverse effects, and perceived reasons for SM among adults in Bangladesh. Participation in the study was voluntary, and individuals willingly participated in the survey. The data collection was carried out from April to June 2021. This study was conducted according to the International Conference of Harmonization (ICH) guidelines for good clinical practice [25].

### 2.2. Study Area

The study was carried out in two administrative districts, Kurigram and Comilla, found in the Northern and Eastern regions of Bangladesh, respectively. The districts are located at 337 km and 100 km from Dhaka, the capital of Bangladesh, respectively. The districts were chosen based on the accessibility and availability of the participants.

### 2.3. Study Tools and Data Collection

An interviewer-administered questionnaire was used to collect data from the participants. The draft version of the questionnaire was pre-tested on 50 adults from the Kurigram district to eliminate confusing and unclear items that were not included in the final analysis. For example, we removed independent variables such as decision-maker, access to healthcare services, and past medical history. The questionnaire was structured and adopted from recent existing literature [26]. A pilot study was conducted with a sample of 20 adults to test the draft version of the questionnaire. The purpose was to identify any unclear or ambiguous items and to determine the time required for the interview. After analyzing the results of the pilot questionnaire, adjustments were made to certain variables because test participants pointed out that some questions or statements were unclear. For example, we modified several questions related to the independent variables, such as self-rated health status, healing methods, and disease names. To evaluate the research themes, the questionnaire was divided into three domains, including sociodemographic and clinical information, indications and reasons for SM, and disease conditions and the presence or absence of adverse effects of SM. The data were collected by trained interviewers after receiving consent from the participants. Any questions and doubts of the interviewers were communicated quickly face-to-face by the principal investigator. The interview took approximately 20–25 min for each participant.

### 2.4. Sampling and Sample Size

Participants who met inclusion criteria (Bangladeshi by birth, lived at least six months in the selected districts, and aged > 19 years) were recruited via home visits. Participants were recruited through systematic door-to-door home visits across various neighborhoods within the selected districts. During these visits, researchers made sure to wear name tags that clearly showed their affiliation and purpose. They also took the time to explain to potential participants the objectives, procedures, and voluntary nature of the study. The recruitment process focused on comprehensive coverage of designated areas, ensuring representation across different neighborhoods. In addition, researchers followed a convenience sampling strategy, approaching households sequentially without deliberately skipping any, while driving around neighborhoods to ensure a diverse and inclusive sample composition. We excluded individuals who were critically ill, incapable of hearing and speaking during the survey, and absent from the home during data collection. The sample size was determined by the single proportion formula (n = Z^2^ × P (1 − P)/d^2^) using the following assumptions: 95% confidence level (1.96), 5% margin of error, and 73.6% SM extent in Bangladesh [13]. Based on the equation, the final required sample size was 300. However, we included 1320 participants to reach a more accurate conclusion in our research.

### 2.5. Outcomes and Covariates

Any SM that took place in the 3 months before conducting the interview with responses denoting “self-medication” or “no self-medication” was used as a dependent variable. This methodology was implemented to address the recall bias frequently observed in retrospective studies, [27]. SM refers to the independent selection and application of over-the-counter medications by individuals to treat themselves or when they perceive themselves as having disorders or symptoms [26,28]. Ailments included in the assessment encompassed gastric issues (gas trouble, acidity), digestive system disorders (diarrhea, vomiting, constipation), common cold/flu (including cough), anxiety/depression, fever, headache, asthma, allergies, weakness, high blood pressure, ear problems, skin problems (acne, pimples, or other), eye infections, tooth pain, and insomnia. The covariates are socio-demographic factors such as gender, age (age were classified as ≤30, 31–60, and >60 years), year of schooling, marital status, occupation, household income, residence, number of family members, and religion. The major health-related factors were coverage of health insurance, health status checks every six months (assessments conducted to monitor an individual’s overall health and well-being), present illness (any ongoing health conditions or ailments experienced by participants at the time of the study), and most-used treatment modality. In addition, data about current smoking was assessed by asking the question “Do you currently smoke any tobacco products, such as cigarettes?”. Knowledge of drug use, membership of a social group (membership in NGOs, service groups, and community welfare organizations), and current treatment status were also used. Participants were asked to self-report their knowledge of drug use, based on their own perceptions or experiences, rather than being observed or tested directly. Knowledge of drug use was self-reported, with options of “none,” “limited,” “somewhat good,” and “excellent.” To facilitate the interpretation of the results, drug use knowledge was reclassified as a dichotomous variable: inadequate knowledge of drugs (a combination of “none” and “limited”), and adequate knowledge of drugs (a combination of “somewhat good” and “excellent”).

### 2.6. Statistical Analysis

Before analyzing the dataset, data cleaning and management were performed. The survey data were analyzed using IBM SPSS version 28.0. Simple descriptive analyses were used to determine the frequencies, percentages, and mean ±SD to quantify different variables. Then, Chi-square and Fisher’s exact tests were conducted to identify the significant differences among variables. Multicollinearity was checked by a correlation coefficient, and a cut-off value of 0.80 was used to select independent variables. Next, both the bi-variable and multi-variable logistic regression analyses were performed to examine the factors associated with SM by our selected independent variables. The logistic regression was validated by the Hosmer–Lemeshow test (χ^2^ = 10.17, 10 df, *p* = 0.252). The adjusted odds ratio (AOR) at 95% confidence intervals was used to measure association (*p*-value < 0.05. A forest plot was used for the graphical display of the significant findings.

## 3. Results

The socio-demographic profiles of the respondents are presented in Table 1. A total of 1320 respondents agreed to participate in this study (1680 doors were knocked on, resulting in various responses including refusals, ineligibility, and non-contacts due to absenteeism), with a response rate of 78.6%. About 50.9% of participants were male, 69.3% were ≤30 years old, and 87.8% were from rural areas. Most participants (71.1%) had incomes of <15,000 BDT a month; however, only 6% were covered by health insurance.

Despite having inadequate knowledge of the drugs involved, just less than half of respondents chose SM. A large number (98.3%) of respondents reported not having adequate knowledge of the drug used. During the study, less than half (35.8%) of the respondents were under treatment by a doctor. The extent of SM was 51.2% among male respondents and 31% among female respondents. The overall prevalence of SM was 41.3%. Among the study participants, the highest SM was observed in businessmen (74%), 72.8% in those who had >12 years of schooling, followed by 63.6% in the respondents who had household income >30,000–50,000 BDT.

The SM of respondents were significantly associated with a host of socio-demographic factors including gender (χ^2^ = 55.38, *p* = 0.001), age (χ^2^ = 16.22, *p* = 0.001), years of schooling (χ^2^ = 43.31, *p* = 0.001), occupation (χ^2^ = 107.41, *p* = 0.001), household income (χ^2^ = 20.57, *p* = 0.001), residence (χ^2^ = 12.30, *p* = 0.001), religion (χ^2^ = 5.07, *p* = 0.024), and family members (χ^2^ = 24.83, *p* = 0.001) and with clinical factors like smoking status (χ^2^ = 29.35, *p* = 0.001), health insurance (χ^2^ = 14.91, *p* = 0.001), membership of a social group (χ^2^ = 34.65, *p* = 0.001), regular check-ups (after every 6 months) (χ^2^ = 24.14, *p* = 0.001), present illness (χ^2^ = 19.33, *p* = 0.001), knowledge of drug used (χ^2^ = 5.53, *p* =0.019) under treatment (χ^2^ = 19.33, *p* = 0.001), and treatment modality (χ^2^ = 37.24, *p* = 0.001) (Table 1).

**Figure 1:**
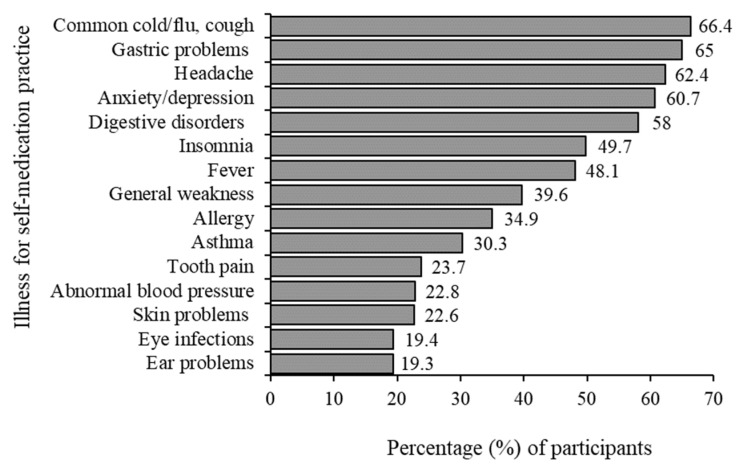
Symptoms that patients self-medicated.

The medical reasons for seeking treatment are largely common ailments. The most frequently found illnesses treated by SM were common cold/flu, cough (66.4%), and gastric problems (65%), followed by headache (62.4%), anxiety/depression (60.7%), and insomnia (50%). The lowest levels of SM were associated with eye infections (19.4%) and ear problems (19.3%), abnormal blood pressure (22.8%), and skin problems (22.6%) (Figure 1).

**Figure 2:**
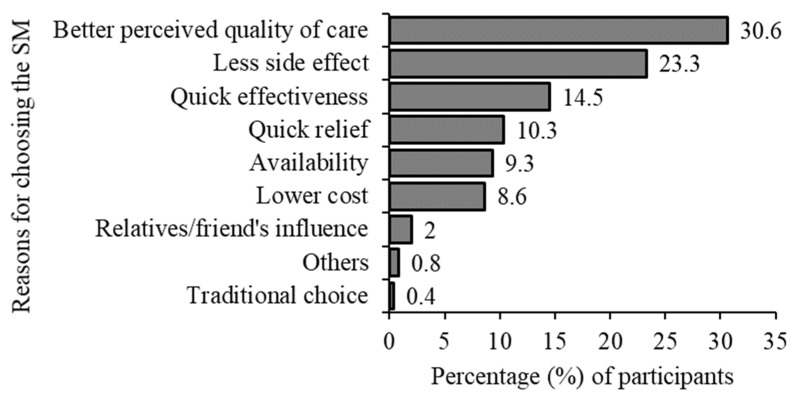
Reasons for choosing self-medication.

We found that quality of care issues topped the reasons for choosing SM, with “better perceived quality of care” being the most common reason for choosing SM and “with less adverse side effects” being the second (30.6% and 23.3%, respectively). Better perceived quality of care included quality of care provided by healthcare practitioners, the accessibility and convenience of healthcare facilities, and the overall patient experience during SM. Next, we found that immediacy issues were reported, and the responses “rapid effectiveness” (14.6%) and “quick relief” (10.3%) were common reasons for choosing SM (Figure 2).

Among respondents, SM was found to be highly successful. The self-reported relief rate (93%) (Table 2) among those choosing SM was significant and 61.9% of respondents did not find any adverse effects in practicing SM (Table 2). Only 3.1% of respondents reported experiencing side effects from SM, while the vast majority (96.9%) did not experience any side effects (Table 2).

**Figure 3:**
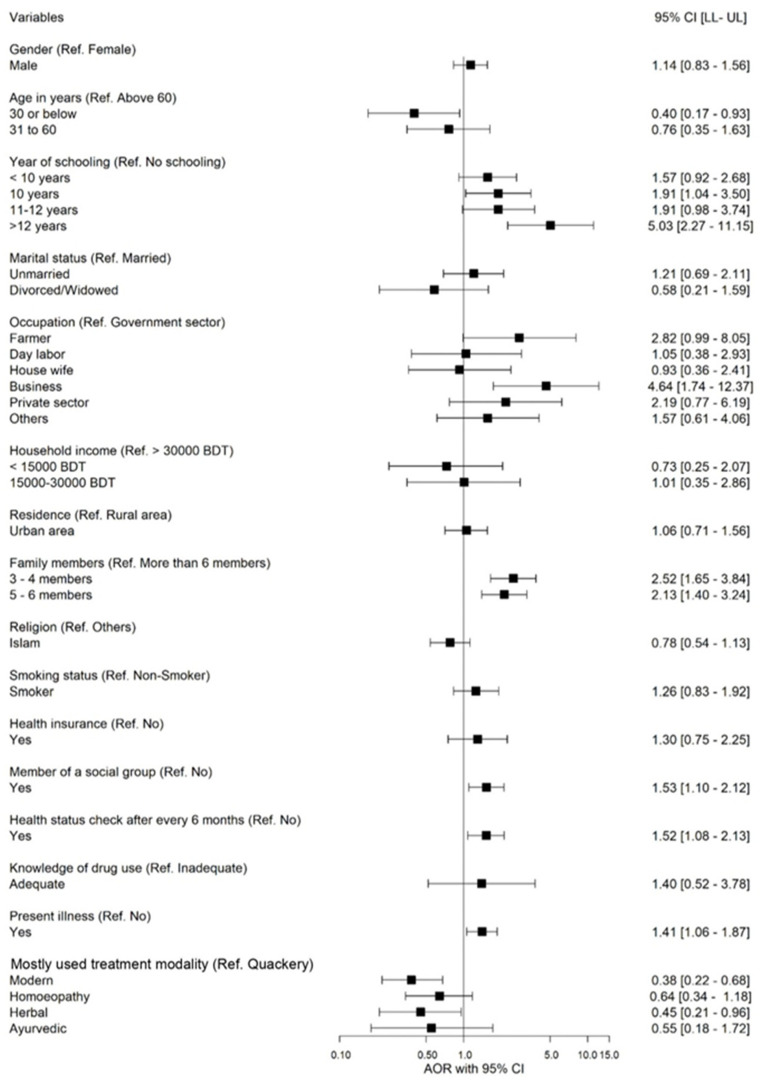
Forest plot of odds ratios and 95% CI representing the correlation between predictors and practice of self-medication.

We performed univariable and multivariable logistic regression analysis and regressed socio-economic and clinical factors upon SM (Table 3 and Figure 3). The factors significantly associated with SM included age, years of schooling, occupation, family members, membership of a social group, health status check, present illness, and treatment modality. In this study, the younger age group (≤30 years) had practiced approximately 60% less SM (AOR = 0.40; 95% CI: 0.17–0.93) compared to the older age group (>60 years). The respondents with > 12 years of schooling had a more than five times higher chance of practicing SM than those without schooling (AOR = 5.03; 95% CI: 2.27–11.15). Those who worked in private business had 4.64 times (AOR = 4.64; 95% CI: 1.74–12.37) higher SM chance than those involved in government service.

Similarly, families having 3 to 4 family members or 5 to 6 family members had 2.52 times (AOR = 2.52; 95% CI: 1.65–3.84) and 2.13 times (AOR = 2.13; CI: 1.40–3.24) higher chances to practice SM, respectively. Among the respondents, those who were members of a social group were 1.53 times more likely to have SM (AOR = 1.53; 95% CI: 1.10–2.12) behavior than those not engaged with a social group. Likewise, respondents who checked their health status (after every 6 months) were 1.52 times more likely to practice SM than those who did not check their health status (AOR = 1.52; 95% CI: 1.08–2.13), and who had present illness were 1.41 times more likely to practice SM (AOR = 1.41; 95% CI: 1.06–1.87). This study also found that those who use modern treatment and herbal treatments as their treatment modality were approximately 62% (AOR = 0.38; 95% CI: 0.22–0.68) and 55% (AOR: 0.45; 95% CI: 0.21–0.96) less likely to practice SM than quackery, respectively.

## 4. Discussion

According to the current study, SM among Bangladeshi adults is substantial at 41.3%. This discovery aligns with a previous study in Bangladesh, which reported a prevalence of 49.9% [29], and a study in Gondar town, Northwest Ethiopia, with a similar rate of 50.2% [30]. Our findings show a higher prevalence of SMP compared to studies carried out in the Meket district, Northeast Ethiopia (35.9%) [30], Eastern Ethiopia (15.8%), India (32.5%) [9], and Nepal (38.2%) [11]. Contrary to that, the SMP in our study (41.3%) is lower compared to studies in Bangladesh (60.2%) [14], Malaysia (62.7%) [31], and Pakistan (84.8%) [10]. The variations in SMP prevalence underscore how SM choices are impacted by demographic factors like cultural beliefs, social determinants of health, variation in recall periods used in every study, socioeconomic gaps, settings, and law enforcement. The current study offers valuable insights into the prevalence of SM in Bangladesh, giving a nuanced perspective compared to national and international studies.

Our research shows that the most common reasons for SM were the common cold/flu, cough, and headache, with gastric issues also playing a significant role. This aligns with a study conducted in Ethiopia among clients visiting community pharmacies [32]. In addition, our findings closely resemble previous research, where fever, body aches, common cold, headache, and cough were identified as significant reasons for SM [33,34,35], including cough and cold remedies, pain relief drugs, diarrhea treatments, allergy medications, constipation remedies, weight loss products, and heartburn medications [31]. The difference in common diseases that result in SM could be due to the varying prevalence of disease in different countries, highlighting the impact of cultural, environmental, and healthcare system factors on SM.

This study highlights the most frequent reasons for choosing SM, including improving service quality, reducing side effects, and saving time, as perceived by consumers. These reasons are commonly linked to the perceived effectiveness of SM practices in addressing health concerns. In Ethiopia, households cited reasons such as the severity of illnesses, emergency cases, reducing medical costs, lack of trust in prescribers, and saving time [30]. In India, the common reasons for SM include saving time and not considering the condition severe enough to require a doctor’s visit [33]. The current study found that a small percentage (3.1%) experienced adverse effects after SM. This aligns with earlier studies, where 28.2% of the subjects experienced adverse effects from the medication [36]. The comparatively low incidence of reported adverse effects may be due to factors beyond participants’ awareness or carefulness. For example, certain modalities, such as homeopathy, may have fewer side effects than traditional treatments. This emphasizes the significance of further research into the factors that influence SM in Bangladesh.

The current study revealed that the older age group is more inclined towards SM than other age groups regarding associated factors. This finding is consistent with a similar survey conducted in Brazil [37] but contrasts with previous research conducted in Ethiopia, which found that the younger age group was more inclined towards SM [32]. However, some other studies reported no association between age and SM [26,38]. The possible reason for the higher prevalence of SM among older adults is the elevated risk of various diseases at an older age. Also, comorbidity in this age group can contribute to increased practice of SM [39], which may need to be resolved with special attention.

In the present study, participants with higher educational attainment practice more SM. This finding agrees with the study conducted in India [40], and several studies also underlined the fact that education level is the dominant factor for the practice of SM [28], but this differed in the Ethiopian sample [38]. Higher-educated individuals may be more inclined to self-medicate due to their knowledge base and ability to access information online. It is crucial to consider that the influence of education on SM behaviors may differ based on the cultural setting and healthcare system of the respondents’ countries [41]. While primarily descriptive in nature, our study contributes to the growing body of literature by shedding light on this relationship and highlights the importance of tailored interventions for promoting responsible self-care in Bangladesh.

Our findings indicate that SM among businessmen was 4.64 times higher than that of the respondents who are government sector workers. This finding is strongly supported by the study in India [40]. While limited time for consultation with health professionals due to hectic schedules or other constraints may contribute to increased SM rates among businessmen, more research is needed to investigate the underlying factors behind this association.

According to this study, families with less than six members were more inclined to engage in SM frequently. This could indicate a correlation between family size and SM. Smaller families may have reasons for utilizing SM more regularly, such as limited resources or access to healthcare. In another study conducted in Ethiopia, no clear connection was found between family size and SM [30]. This emphasizes the significance of considering cultural and regional factors when analyzing this relationship. Further research across diverse settings could provide valuable insights into why families practice SM. According to the current study, there is a link between social group participation and higher practice of SM [42]. However, it is critical to emphasize that this finding lacks direct evidence from the available literature and must be validated by additional studies. Furthermore, our study revealed that responders who receive frequent medical check-ups every six months practice more SM. While regular check-ups are important for maintaining health and recognizing potential concerns early on, a previous study found contrary results, indicating a negative association between routine check-ups and SM practice [41]. This disparity highlights the complexities of the link between health-seeking behaviors, routine check-ups, and SM. It emphasizes the need to consider various factors, including cultural nuances, healthcare system structures, and individual attitudes, when understanding the dynamics of SM across contexts.

Quacks (persons unauthorized to practice medicine) of Bangladesh usually do not charge consultation fees; medicine-selling profit is their primary source of income [43]. People from rural areas with lower income might prefer the treatment of quacks because of their poverty [43]. The current study revealed that people using quackery as a treatment modality practice more SM. It could be explained by the fact that quacks frequently exploit distrust of the medical system. Additionally, if a person has had a terrible encounter with a doctor or does not comprehend medical science, they may be more prone to believe a quack’s claims and take action themselves. Moreover, quacks often promise quick and easy solutions, but if not fulfilled, people might become frustrated and resort to self-treating with readily available substances or following unproven remedies. Furthermore, quacks often lack proper guidance and focus on selling their products, leading to people experimenting with SM without appropriate diagnosis and treatment plans [44].

It is difficult to find fault with decisions that seem logical, and while education often helps to discourage improper behavior, it encourages it here. They are using sound reasoning and find the likelihood of negative outcomes of SM to be low. However, they concluded that the small likelihood of a negative event means the risk of SM is low. These well-meaning individuals are not properly considering the potential risks of missed or incorrect diagnosis, potential drug interactions, or effects of counterfeit drugs, which, though rare, may be catastrophic. Taking their medical treatment into their own hands puts them and their communities at risk for events of low frequency but substantial severity.

This study has several limitations, and we confronted some constraints. Firstly, this study used a cross-sectional design; therefore, it cannot prove causality. Secondly, we assessed only common illnesses like headache, common cold/flu, cough, anxiety/depression, etc., to determine SM and exclude contagious diseases like COVID-19, norovirus, sexually transmitted infections (STIs), etc., from our study. Thirdly, we did not collect data on drugs used and the duration of drugs used for a specific disease. Fourthly, while our study provides vital insights into SM practices in the sampled community, it is important to take caution when generalizing the findings to the total population of both cities. Additionally, there was very limited research on this emerging topic to carry out any final conclusion. Moreover, we collected data from two selected districts of Bangladesh. Therefore, the prevalence of SM in other districts might be different. Finally, this study had a limited age group of people as respondents, and we did not collect data for adolescents or children for SM.

## 5. Conclusions

The main finding of this study was that nearly half of adults practiced SM in Bangladesh. Several factors were associated with SM, for example, years of schooling, being a businessman, having larger family members, being a member of a social group, checking health status every six months, older adults, present illness, and using quackery as a treatment modality. Therefore, people should be educated on the potential risks of SM. Moreover, the general public needs to be aware of the differentiation between certified medical practitioners and quacks. To mitigate the potential risk of SM, healthcare policymakers should promote awareness programs on the rational use of medications and enforce laws to dispense prescription medication by registered physicians.

## Figures and Tables

**Table 1:** Association of socio-demographic and clinical variables with self-medication practice.

Variables	Total N = 1320		Self-Medication				χ^2^	*p*-Value
			No		Yes			
	n	%	n	%	n	%		
Gender								
Male	672	50.9	328	48.8	344	51.2	55.38	<0.001
Female	648	49.1	447	69.0	201	31.0		
Age in years								
≤30	915	69.3	569	62.2	346	37.8	16.22	<0.001
31 to 60	361	27.3	180	49.9	181	50.1		
>60	44	3.3	26	59.1	18	40.9		
Years of schooling								
<10 years	510	38.6	315	61.8	195	38.2	43.31	<0.001
10 years	425	32.2	254	59.8	171	40.2		
11–12 years	172	13.0	100	58.1	72	41.9		
>12 years	92	7.0	25	27.2	67	72.8		
No schooling	121	9.2	81	66.9	40	33.1		
Marital status								
Unmarried	682	51.7	406	59.5	276	40.5	1.09	0.580
Divorced/widowed	21	1.6	14	66.7	7	33.3		
Married	617	46.7	355	57.5	262	42.5		
Occupation								
Farmer	63	4.8	23	36.5	40	63.5	107.41	<0.001
Day labor	76	5.8	50	65.8	26	34.2		
Housewife	307	23.3	231	75.2	76	24.8		
Business	104	7.9	27	26.0	77	74.0		
Private	49	3.7	18	36.7	31	63.3		
Others ^a^	690	52.3	412	59.7	278	40.3		
Government	31	2.3	14	45.2	17	54.8		
Household income per month								
<15,000 BDT	938	71.1	586	62.5	352	37.5	20.57	<0.001
15,000–30,000 BDT	360	27.3	181	50.3	179	49.7		
>30,000 BDT	22	1.7	8	36.4	14	63.6		
Residence								
Urban area	161	12.2	74	46.0	87	54.0	12.30	<0.001
Rural area	1159	87.8	701	60.5	458	39.5		
Religion								
Islam	1156	87.6	692	59.9	464	40.1	5.07	0.024
Others ^b^	164	12.4	83	50.6	81	49.4		
Family members								
3–4 members	592	44.8	322	54.4	270	45.6	24.83	<0.001
5–6 members	553	41.9	321	58.0	232	42.0		
Above 6 members	175	13.3	132	75.4	43	24.6		
Smoking status								
Smoker	187	14.2	76	40.6	111	59.4	29.35	<0.001
Non-smoker	1133	85.8	699	61.7	434	38.3		
Health insurance								
Yes	79	6.0	30	38.0	49	62.0	14.91	<0.001
No	1241	94.0	745	60.0	496	40.0		
Member of a social group								
Yes	249	18.9	105	42.2	144	57.8	34.65	<0.001
No	1071	81.1	670	62.6	401	37.4		
Health status check (after every 6 months)								
Yes	223	16.9	98	43.9	125	56.1	24.14	<0.001
No	1097	83.1	677	61.7	420	38.3		
Knowledge of drug used								
Adequate	23	1.7	8	34.8	15	65.2	5.53	0.019
Inadequate	1297	98.3	767	59.1	530	40.9		
Present illness								
Yes	483	36.6	246	50.9	237	49.1	19.02	<0.001
No	837	63.4	529	63.2	308	36.8		
Under treatment								
Yes	473	35.8	240	50.7	233	49.3	19.33	<0.001
No	847	64.2	535	63.2	312	36.8		
Most frequent treatment modality								
Modern	873	66.1	563	64.5	310	35.5	37.24	<0.001
Homeopathy	284	21.5	139	48.9	145	51.1		
Herbal	69	5.2	34	49.3	35	50.7		
Ayurvedic	19	1.4	8	42.1	11	57.9		
Quackery	75	5.7	31	41.3	44	58.7		

Note: ^a^ = includes fishermen, garments workers, vehicles driver, swipers, and unemployed ^b^ = includes Hindu, Christian, Buddhist.

**Table 2:** Disease conditions and side effects resulting from self-medication.

Variables	Frequency	Percentage
Diseases conditions after self-medication		
Not relieved	28	5.1
Relieved	507	93
Don’t know	10	1.9
Side effects of self-medication		
No	528	96.9
Yes	17	3.1

**Table 3:** Bivariate analysis of the factors affecting self-medication practice.

Variables and Categories	*p*-Value	COR 95% CI [LL–UL]
Gender Ref. (Female)		
Male	<0.001	2.33 [1.86–2.92]
Age (Ref. >60 Years)		
≤30 Years	0.680	0.88 [0.48–1.63]
31 to 60 years	0.250	1.45 [0.77–2.74]
Years of schooling (Ref. No schooling)		
< 10 years	0.290	1.25 [0.83–1.91]
10 years	0.153	1.36 [0.89–2.09]
11–12 years	0.128	1.46 [0.90–2.37]
>12 years	<0.001	5.43 [2.99–9.84]
Marital status (Ref. Married)		
Unmarried	0.466	0.92 [0.74–1.15]
Divorced/widowed	0.407	0.68 [0.27–1.70]
Occupation (Ref. Government sector)		
Farmer	0.420	1.43 [0.60–3.43]
Day labor	0.051	0.43 [0.18–1.00]
Housewife	0.001	0.27 [0.13–0.58]
Business	0.044	2.35 [1.02–5.40]
Private sector	0.454	1.42 [0.57–3.54]
Others	0.111	0.56 [0.27–1.15]
Household income (Ref. >30,000 BDT)		
<15,000 BDT	0.017	0.34 [0.14–0.83]
>15,000–30,000 BDT	0.210	0.57 [0.23–1.38]
Residence (Ref. Rural area)		
Urban area	0.001	1.80 [1.29–2.51]
Family members (Ref. More than 6 members)		
3–4 members	<0.001	2.57 [1.76–3.77]
5–6 members	<0.001	2.22 [1.51–3.26]
Religion (Ref. Others)		
Islam	0.025	0.69 [0.50–0.95]
Smoking status (Ref. Non-smoker)		
Smoker	<0.001	2.35 [1.72–3.23]
Health insurance (Ref. No)		
Yes	<0.001	2.45 [1.54–3.92]
Member of a social group (Ref. No)		
Yes	<0.001	2.29 [1.73–3.03]
Health status check (after every 6 months) (Ref. No)		
Yes	<0.001	2.06 [1.54–2.75]
Knowledge of drug used (Ref. Inadequate)		
Adequate	0.024	2.71 [1.14–6.45]
Present illness (Ref. No)		
Yes	<0.001	1.66 [1.32–2.08]
Most frequent treatment modality (Ref. Quackery)		
Modern	<0.001	0.39 [0.24–0.63]
Homeopathy	0.241	0.74 [0.44–1.23]
Herbal	0.339	0.73 [0.38–1.40]
Ayurvedic	0.951	0.97 [0.35–2.69]

Note: COR: crude odds ratio, CI: confidence interval, LL: lower limit, UL: upper limit.

## Data Availability

The data of the study are available upon request to the corresponding author.
